# Stocking Practices of Anti-Tuberculosis Medications among Community Pharmacists and Patent Proprietary Medicine Vendors in Two States in Nigeria

**DOI:** 10.3390/healthcare11040584

**Published:** 2023-02-15

**Authors:** Victor Abiola Adepoju, Ademola Adelekan, Olanrewaju Oladimeji

**Affiliations:** 1 Department of HIV and Infectious Diseases, Jhpiego (An Affiliate of John Hopkins University), Abuja 190918, Nigeria; 2 Blue Gate Research Institute, Ibadan 200285, Nigeria; 3 Department of Public Health, Faculty of Health Sciences, Walter Sisulu University, Mthatha 5099, South Africa; 4 Faculty of Health Sciences, Durban University of Technology, Durban 4001, South Africa

**Keywords:** tuberculosis, patent medicine vendors, community pharmacists, anti-TB medication, stocking, dispensing, fixed-dose combination

## Abstract

Background: Evidence has shown that non-fixed-dose combination (non-FDC) anti-TB drugs could promote the spread of drug-resistant tuberculosis (DR-TB). We aimed to determine anti-TB medication stocking and dispensing practices among patent medicine vendors (PMVs) and community pharmacists (CPs) and their determinants. Method: This was a cross-sectional study using a structured, self-administered questionnaire among 405 retail outlets (322 PMVs and 83 CPs) across 16 Lagos and Kebbi local government areas (LGAs) between June 2020 and December 2020. Data were analyzed with Statistical Program for Social Sciences (SPSS) for Windows version 17 (IBM Corp., Armonk, NY, USA). Chi-square test and binary logistic regression were used to assess the determinants of anti-TB medication stocking practices at a p-value of 0.05 or less for statistical significance. Results: Overall, 91%, 71%, 49%, 43% and 35% of the respondents reported stocking loose rifampicin, streptomycin, pyrazinamide, isoniazid and ethambutol tablets, respectively. From bivariate analysis, it was observed that being aware of directly observed therapy short course (DOTS) facilities (OR 0.48, CI 0.25–0.89, *p* < 0.019) and having previous training on TB (OR 0.32, CI 0.14–0.73, *p* < 0.005) reduced the odds of stocking anti-TB medication, while operating more than 1 shop (OR 3.32, CI 1.44–7.57, *p* = 0.004), having 3 or more apprentices (OR 5.31, CI 2.74–10.29, *p* < 0.001) and seeing over 20 clients/day (OR 3.02, CI 1.18–7.71, *p* = 0.017) increased the odds of stocking loose anti-TB medications. From multivariate analysis, it was observed that only the variable having three or more apprentices (OR 10.23, CI 0.10–0.49, *p* = 0.001) significantly increased the odds of stocking anti-TB medications. Conclusions: The stocking of non-FDC anti-TB medications was high and largely determined by the number of apprentices among PMVs and CPs in Nigeria, and this may have serious implications for drug resistance development. However, the results linking the stocking of anti-TB to the number of apprentices should be interpreted cautiously as this study did not control for the level of sales in the pharmacies. We recommend that all capacity-building and regulatory efforts for PMVs and CPs in Nigeria should include not just the owners of retail premises but also their apprentices.

## 1. Introduction

Nigeria is a high-burden country for tuberculosis (TB), multidrug-resistant tuberculosis (MDR-TB) and TB/HIV [1,2,3]. In 2018, Nigeria ranked first in Africa and sixth globally among countries with the highest burden of TB [1]. Only 138,591 of the 452,000 patients projected to have TB in 2020 were notified, meaning that 75% of TB cases were neither diagnosed nor notified to the National TB Program (NTP) [1]. Annually, Nigeria accounts for 9% of TB cases missed globally, either because they are not diagnosed, or they are diagnosed but not reported to the National TB, Buruli Ulcer and Leprosy Control Program (NTBLCP) [4]. The private sector has been acknowledged globally as having the potential to accelerate TB control efforts and find missing TB cases. Evidence shows that most healthcare service provisions for presumptive TB clients occur in CPs and PMVs, as they represent the first port of care for individuals with presumptive TB symptoms such as cough, fever, night sweats or weight loss; hence, they are important for TB control efforts in low- and middle-income countries (LMICs) [5,6]. Currently, CPs and PMVs are engaged in Nigeria by the NTBLCP for the symptomatic screening, identification and referral of presumptive TB patients to DOTS facilities affiliated with the NTBLCP. The number of PMVs was estimated to be 200,000 in 2005, roughly 100 times greater than the number of registered CPs and nearly 4 times the number of physicians [7]. Although both PMVs and CPs are medicine vendors, PMVs receive informal health training and can only stock limited medications approved by the Pharmacists Council of Nigeria (PCN), while CPs receive formal university education in pharmacy and stock a wider range of drugs over the counter [8]. Anti-TB stocking practices in this context refer to the methods used to ensure that adequate supplies of anti-tuberculosis (TB) drugs are available for dispensing in PMV or CP stores and whether stocking and dispensing practices are in line with the regulations of the NTBLCP. The NTBLCP is responsible for the procurement of quality-assured fixed-dose combinations either directly or through the global drug facility. Fixed-dose anti-TB medications are supplied to public and private facilities affiliated with the NTBLCP, while PMVs and CPs identify and refer presumptive TB clients from their shops to NTBLCP-affiliated facilities for TB confirmatory diagnosis and treatment. One of the goals of NTBLCP is to reduce the spread of drug-resistant TB (DR-TB) from the sales and use of loose, non-FDC tuberculosis drugs. First-line drugs are usually administered in fixed-dose combinations (FDCs), which are combination tablets that contain two or more drugs in a single tablet. Some examples of first-line FDCs are rifampicin, isoniazid, pyrazinamide and ethambutol. However, rifampicin, isoniazid, pyrazinamide and ethambutol can also be administered as individual, loose tablets, which would make them non-FDCs. In this case, the patient would take each drug separately rather than in a single, combined tablet. The specific treatment regimen, including the formulation of the drugs, is usually determined by a physician based on the individual patient’s circumstances and the severity of their TB disease.

Exposure to single, loose anti-TB drugs (monotherapy) could fuel DR-TB and reverse the gains of national tuberculosis control efforts. CPs are generally perceived to offer a better quality of malaria services than PMVs, although there is limited empirical evidence to support this [9]. One of the risk factors for the development of DR-TB includes the inappropriate use of non-FDC anti-TB regimens. Data from countries such as Bolivia, Nepal and Georgia have shown over-the-counter stocking of non-FDC anti-TB drugs in private pharmacies [10,11,12], and these are believed to fuel the spread of DR-TB. Given the paucity of data highlighting this issue in Nigeria, we aimed to assess anti-TB medication stocking and dispensing practices among PMVs and CPs and their determinants.

## 2. Materials and Methods

### 2.1. Study Design

This is a cross-sectional study using a structured self-administered questionnaire among 405 retail outlets (322 PPMVs and 83 CPs) and across 16 Lagos and Kebbi LGAs (309 from Lagos and 96 from Kebbi state) between June 2020 and December 2020.

### 2.2. Study Setting

Lagos and Kebbi states are located in Southern and Northern Nigeria, respectively. The two states are a holistic representation of the country. Lagos is the commercial center of Nigeria with a population of over 20 million. Lagos is divided into 20 LGAs, the administrative unit below the state [13]. In Lagos, 16 of the 20 LGAs are classified as urban and 4 as rural (Ikorodu, Badagry, Epe and Ibeju Lekki). Kebbi state is in the northern part of Nigeria with its capital Birnin Kebbi and a population of 352,000 in 2019. The predominant language is Hausa and Fulani, while farming is the major occupation [14,15]. Compared to Lagos State, Kebbi is largely rural with a huge population of individuals with lower socioeconomic status. In both states, informal providers are a major source of health service delivery. Common languages in Lagos include English and Pidgin, while Hausa is prominently spoken in Kebbi state. A total of 16 LGAs, 13 in urban Lagos and 3 in rural Kebbi, were selected for this study. These LGAs also have a high TB burden. Health service delivery in Nigeria is provided at primary, secondary and tertiary levels. CPs and PMVs are engaged by the TB control program for the identification and referral of presumptive TB clients to public and private health facilities in order to assess diagnostic and TB/DOTS treatment services.

### 2.3. Tiered Accreditation Approach by the Pharmacy Council of Nigeria

The current pharmacy education system in Nigeria leads to the award of a Bachelor of Pharmacy (B.Pharm) degree after 5 years of study. However, the Pharmacists Council of Nigeria (PCN) has recently shown interest in introducing the Doctor of Pharmacy (PharmD) program as a way to enhance the professional development of pharmacists in Nigeria. The Pharmacists Council of Nigeria has adopted a tiered accreditation approach for PMVs, where they are categorized into three tiers based on their health qualifications. Each tier is authorized to practice service provisions consistent with their qualifications. This system recognizes the background educational qualification(s) that PMVs have and categorizes them into 3 tiers [16], namely:

Tier 1: Tier 1 PMVs (i.e., PMVs lacking health qualifications and any training). They must be able to read and write and have attained 21 years of age.

Tier 2: Tier 2 PMVs (i.e., health-qualified PMVs) must fulfill the tier 1 eligibility criteria for age. In addition, they must possess a tertiary qualification (e.g., bachelor’s or post-secondary diploma) in a health discipline, as recognized by the Pharmacists Council of Nigeria. They could be nurses, midwives, community health extension workers (CHEWs), community health officers (CHOs), etc.

Tier 3: Tier 3 PMVs are pharmacy technicians.

Community pharmacists are pharmacy professionals with tertiary qualifications in pharmacy, which can be in B.Pharm. They are outside of the tiered accreditation system.

### 2.4. Study Size

A total of 405 community pharmacists and patent medicine vendors were included in this study. We calculated sample size using the formula n = a^2^b/d2, where n is the sample size, a is the Z statistic for a level of confidence, b is prevalence and d is precision or confidence interval. The level of confidence of 95% is conventional at which the value for a is 1.96 and d is 0.05. A study in Nigeria previously found that an average of 23% (30.1% in urban and 15.2% in rural areas) of retail stores stocked anti-TB medicines [17]. This equates to a b value of 0.23. We arrived at an approximate sample size of 353 participants. With an assumption of a 20% non-response rate, a total of 405 participants were invited to participate in this study.

### 2.5. Sampling Technique

A 4-stage sampling technique (summarized below) was used to select participants for this study.

Stage 1: Selection of study LGAs.

The purposive sampling technique was used to select 13 LGAs in Lagos and 3 LGAs in Kebbi. These LGAs have a high TB burden and high retail store presence in these states.

Stage 2: Selection of PMVs and CPs.

The list of PMVs and CPs registered in each state in 2019 was requested from relevant professional associations and was stratified into those who were already formally affiliated with and those not formally affiliated with the NTBLCP. The list of PMVs and CPs already affiliated with the NTBLCP was filtered out from the universal list of CPs and PMVs after crosschecking with the list shared by the professional associations.

Stage 3: Selection of PMVs and CPs not formally affiliated with the NTBLCP.

A systematic random sampling approach was used to select the required numbers of participating non-NTP-affiliated CPs and PMVs per LGA and across the 16 LGAs in the two states, proportionate to the number of PMVs and CPs in the LGAs until the sample size was reached.

Stage 4: Selection of PMV and CP shop owners.

The purposive sampling technique was used for selecting the required numbers of CPs and PMVs (shop owners) interviewed in the selected premises. A purposive sample of 322 PMVs and 83 community pharmacy store owners were recruited for this study. The chief pharmacist was selected for the CPs and the chief PMV for the PMVs. Where the chief pharmacist or PMV owner was not available, they were replaced by another attending pharmacist for CP or a PMV assistant who met the inclusion criteria. If more than one CP/PMV met the inclusion criteria, simple random balloting was then used to select respondents to be interviewed.

### 2.6. Participants

Study population included 405 non-NTP-affiliated CPs and PMVs in Lagos and Kebbi states.

Inclusion criteria:

– Age ≥ 18 years;
– PMVs and CPs were licensed by the Pharmacy Council of Nigeria;
– PMVs and CPs were not affiliated with NTBLCP;
– CPs and PMVs were registered with relevant professional associations;
– Having a physical premise where the vendor operates.
– Any CP or PMV who did not meet the inclusion criteria was excluded.

### 2.7. Variables

The outcome/dependent variable was the “stocking of loose anti-TB medications”, categorized into “Stocking anti-TB medications” or “Not stocking anti-TB medications”. Independent variables included participants’ sex, type of business (PMV/CP), highest educational qualification, medical education, years of practice, number of apprentices, clients seen per day, PMV accreditation tier, practice setting, qualification, previous TB training, number of post-graduation years of experience and awareness of DOTS and DOTS facilities. Study variables were presented using descriptive statistics such as frequency and percentage. Associations with anti-TB medication stocking were assessed at a multivariate level using logistic regression. For the type of anti-TB medications that were stocked, categorical responses such as rifampicin, isoniazid, pyrazinamide, ethambutol and streptomycin (RIPES) were presented using frequency and percentage (with multiple responses allowed). Additionally, the frequency of different combinations of anti-TB medications stocked by PMVs and CPs was presented using percentages.

### 2.8. Data Sources and Measure

The questionnaire administered for this study contained 18 questions divided into 2 sections.

Section 1 contained sixteen questions about the sociodemographic information such as sex (M/F), state (Lagos/Kebbi), LGA, type of business provider (PMV/CP), highest educational qualification (primary, secondary, tertiary or none), medical education (Y/N), years of practice (0–5/6 or more), prior TB training (Y/N), number of apprentices (0, 1–2, 3 or more), clients seen per day (1–10, 11–20, 20 or more), referral in the last one month, willingness to be engaged (Yes/No), awareness of DOTS facilities (Yes/No), PMV accreditation tier (1, 2 or 3), kept treatment record (Y/N) and kept referral records (Y/N).

Section 2 asked participants two questions about anti-TB medication stocking practices. These included “Do you currently stock anti-TB drugs (Y/N)” and “If Yes, what type(s) of anti-TB medication do you stock (rifampicin, isoniazid, pyrazinamide, ethambutol and streptomycin)”. A shop was regarded as stocking a particular product if the respondent self-reported that the product was available and dispensed.

### 2.9. Bias

In order to minimize bias, this study comprised private non-NTP-affiliated retail vendors across 16 LGAs from both urban to rural populations in Northern and Southern Nigeria, as well as large- and small-sized CPs and PMVs.

## 3. Data Collection

The researchers received clearance from the relevant professional associations to legitimize their study. The chairmen introduced the researchers to the members and explained the goal. A total of 16 research assistants were trained over 3 days on data collection and GPS, and this study was piloted in 2 LGAs. During data collection, the research assistants explained questions and checked for completion. The questionnaire consisted of two sections on participants’ demographic information and stocking practices of anti-TB medications. The data were reviewed by supervisors and informed consent was taken from participants. This research was conducted from June to December 2020.

### 3.1. Data Analysis

In addressing the research objectives, we applied descriptive statistics (percentages and numbers) to summarize the categorical variables. Sociodemographic variables and types of anti-TB medications stocked (rifampicin, isoniazid, pyrazinamide, ethambutol and streptomycin) were descriptively described. Variables with more than two categories (e.g., tier accreditation, clients seen per day, etc.) were recoded into two categories for the subsequent binary logistic regression model. A bivariate logistic regression model was first fitted, and the variables with a *p*-value < 0.05 in the bivariate were subsequently fitted in the final multivariable logistic regression model. Variables with a *p*-value < 0.05 in the final multivariable logistic regression model were considered significantly associated with the dependent/outcome variable (stocking anti-TB medications, Y/N). Crude and adjusted odd ratios (ORs) with a 95% confidence interval (CI) were calculated to measure the strength of the association between the dependent/outcome (stocking anti-TB medication) and independent variables such as sex, the type of business, highest educational qualification, medical education, years of practice, the number of apprentices, clients seen per day, PMV accreditation tier, practice setting, qualification, previous TB training, the number of post-graduation years of experience and the awareness of DOTS facilities. Some variables have missing information as clients preferred not to answer some questions, e.g., the type of anti-TB drugs stocked. In addressing missing data and their impact on study outcomes, we performed multiple imputations for systematically missing data (i.e., sporadically missing data where variables were available for some data sets but missing for some individuals) using generalized linear mixed models to consider clustering.

### 3.2. Ethical Approval

This study was approved by the Nigerian Health Research and Ethics Committee. Permission (approval code: NHREC/01/01/2007) was also received from the Lagos and Kebbi State Ministry of Health. Respondents’ confidentiality was also maintained by not using identifiers. To anonymize study participants, code numbers rather than personal identifiers were used. Written informed consent was obtained from the respondents prior to the administration of questionnaires.

## 4. Results

In Table 1, it can be seen that the majority of participants were male, 243 (60.4%); from Lagos state, 309 (76.3%); had a tertiary educational qualification, 236 (58.3%); had medical education, 300 (74.1%); spent 6 years or more in practice, 290 (71.6%); were not previously trained on TB, 286 (70.6%); had 1–2 apprentices, 151 (37.3%); consulted over 20 customers/day, 255 (63%); and belonged to PMV tier 1 (a sub-category of PMV) accreditation, 262 (64.7%).

Table 2 compares the stocking pattern of anti-TB medications (RIPES) among PMV and CP. A greater percentage of CPs stocked isoniazid (72.4% vs. 27.4%) and pyrazinamide (51.5% vs. 48.5%), while a greater percentage of PMVs stocked rifampicin (53.2% vs. 46.8%), ethambutol (68.2% vs. 31.8%) and streptomycin (58.3% vs. 41.7%). There was a significant difference in the stocking practices of isoniazid and ethambutol among CPs and PMVs, respectively, as a significantly greater percentage of CPs stocked isoniazid (*p* < 0.001) and a significantly larger percentage of PMVs (*p* = 0.001) stocked ethambutol.

Table 3 shows the anti-TB stocking practices and associated factors. From bivariate analysis, it can be seen that being aware of DOTS facilities (OR 0.48, CI 0.25–0.89, *p* < 0.019) and having been previously trained on TB (OR 0.32, CI 0.14–0.73, *p* < 0.005) reduced the odds of stocking anti-TB medication, while operating more than 1 shop (OR 3.32, CI 1.44–7.57, *p* = 0.004), having 3 or more apprentices (OR 5.31, CI 2.74–10.29, *p* < 0.001) and seeing over 20 clients/day (OR 3.02, CI 1.18–7.71, *p* = 0.017) increased the odds of stocking loose anti-TB medication. From multivariate analysis, it can be seen that only the variable having three or more apprentices (OR 10.23, CI 0.10–0.49, *p* < 0.001) significantly increased the odds of stocking anti-TB medications.

**Figure 1:**
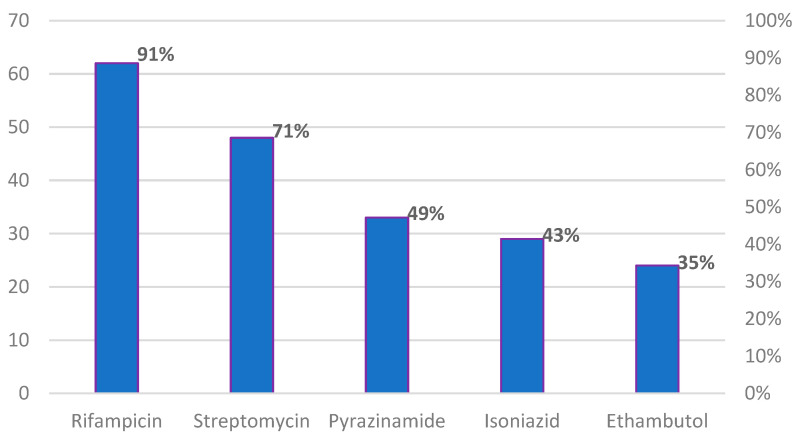
Reported sale of first-line anti-TB drugs for the treatment of cough among PMVs and CPs, n = 68. Multiple responses allowed.

In Figure 1, it can be observed that among the 68 participants that responded yes to the question on stocking of anti-TB medication for the treatment of prolonged cough, 91%, 71%, 49%, 43% and 35% of respondents admitted stocking rifampicin, streptomycin, pyrazinamide, isoniazid and ethambutol, respectively.

**Figure 2:**
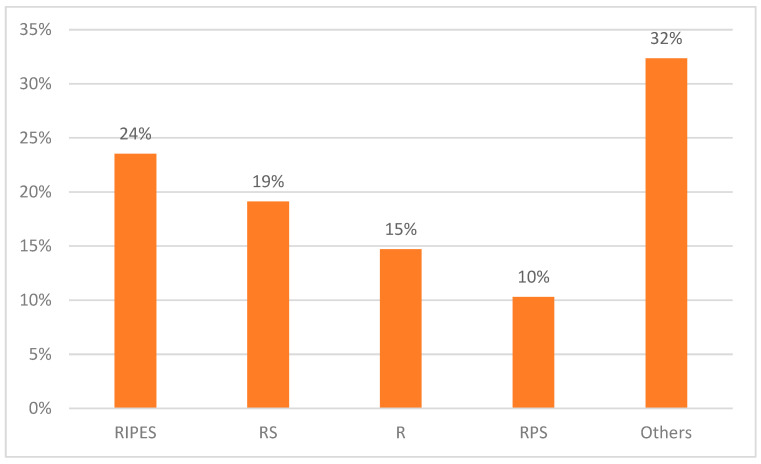
Percentage of CPs and PMVs who reported stocking different loose anti-TB drugs, n = 68.

In Figure 2, it can be observed that 68 participants responded yes to the question on stocking anti-TB medication for the treatment of prolonged cough. There was a total of 20 different types or combinations of anti-TB drugs reported as stocked by CPs and PMVs. Of these, 3 (15%) were single anti-TB drugs (isoniazid, rifampicin and streptomycin), while 17 (85%) reported a combination of anti-TB drugs (e.g., RIPES, RPS, RS, RPES, RES, RI, etc.). CPs and PMVs mostly stocked a combination of RIPES, 16 (24%); followed by RS, 13 (19%); and rifampicin, 10 (15%).

## 5. Discussion

This study aimed to investigate anti-TB medication stocking practices and their determinants among patent medicine vendors and community pharmacists operating drug outlets in Nigeria. We observed from bivariate analysis that being aware of DOTS and having been previously trained on TB reduced the odds of stocking anti-TB medications, while operating more than 1 shop, having 3 or more apprentices and seeing over 20 clients per day increased the odds of stocking anti-TB medication. However, from multivariate analysis, it could be observed that PMVs and CPs with three or more apprentices had significantly increased odds of stocking anti-TB medications. The findings of this study have far-reaching implications on how regulatory institutions, program managers and the Ministry of Health are engaging PMVs and CPs in Nigeria in the context of apprenticeship programs. Apprenticeship is a capacity-building system of a new generation of practitioners using on-the-job training with or without accompanying study in a bid to learn the rudiments of drug supplies, products, sales and pricing, among others [18]. Many PMVs in Nigeria usually complete informal training from a more senior PMV or CP before opening their own shop. Studies have shown that 11.7%, 13.8% and 22% of PMVs encountered in Kaduna, Lagos and Enugu states in Nigeria, respectively, were apprentices [19,20,21]. PMVs are usually managed by apprentices when the shop owners are away, as many owners have concurrent employment serving as multiple sources of income. Most donor-funded vertical programs and public health interventions in Nigeria often engage PMVs and CPs through training workshops and other capacity-building activities [22,23], hence the need to re-imagine the engagement process beyond the licensed/registered shop owners and expand quality assurance efforts to include the apprenticeship programs in light of current study findings. In many situations, apprentices are young and inexperienced and leaving the entire patient management, including stocking, dispensing and sales of medications, to these individuals without the supervision of a licensed operator would be counterproductive to the public health goal of saving lives and improving access to health services in hard to reach communities.

Currently, the Pharmacy Council of Nigeria stipulates mandatory attendance of the orientation program and continuing professional development (CPD) courses as a precondition for license renewal of PMVs and CPs in Nigeria [8]. However, these quality assurance and capacity-building efforts must be extended to the apprentices and sales attendants working on these premises for them to be effective. A previous study that assessed the compliance of PMVs in Nigeria with malaria regulatory guidelines observed that apprentices and sales attendants sold antimalaria drugs despite their limited knowledge of malaria management when compared with shop owners, thus, being unable to sell drugs at appropriate doses, leading to the spread of drug resistance, severe complicated malaria and death [19]. Similarly, in Ghana, training shop owners on patient communication only led to a modest improvement in the proportion of patients fully adherent to an antimalaria regimen, as only the shop owners were trained with the assumption that they would pass down the training to the apprentices working for them [24]. Apprentices could knowingly or unknowingly be stocking loose non-FDC anti-TB tablets while restocking on behalf of their principal due to limited knowledge, hence promoting the spread of multidrug-resistant TB in the community. Several studies have highlighted the challenges and possible reasons for the gaps in the provision of health services in the context of apprenticeship and the support staffing system of CPs and PMVs. These include the lack of competencies of support staff and reluctance to take on greater professional responsibilities; inadequate structured training that delineates administrative support from clinical decisions leading to low self-efficacy; and increasing pressure from pharmacists on apprentices to meet targets, thereby encouraging rule-breaking behaviors that disregard guidelines, policies and procedures [25,26,27,28,29]. Providers may opt for drugs of poor quality for lack of adequate funds to support a quality-centered business model or when such drugs are not readily accessible for wholesale purchase. For instance, in Tanzania, the accredited drug dispensing outlet (ADDO) regulations require drug shops to purchase from registered suppliers, but most districts lack wholesaling pharmacies [30]. The program works with national wholesalers to establish branches in pilot areas, leading to the creation of the Southern Highlands Pharmacy in Songea Urban. ADDOs (especially those in rural areas) are also linked to loan opportunities from microfinance banks and given some tax waivers to boost their businesses. In Kenya, pharmacy support staff are recruited under the retail drug seller educational program as volunteer educators of peers on the new malaria guideline (vendor to vendor) and are provided with awareness posters and job aids, leading to improved anti-malaria stocking practices and enhanced knowledge of anti-malaria prescription. Therefore, ongoing capacity-building and incentive systems that discourage unstandardized practices are critical policy decisions that need to be implemented in order to maximize the values and benefits of community pharmacists and patent medicine vendors in Nigeria.

## 6. Strength and Limitations

This study included a large sample size and diverse participants from both the northern and southern parts of the country (309 from the south and 96 from the north), making the findings generalizable to the entire Nigerian population and similar settings. However, some respondents might have deliberately concealed information on the stocking of non-FDC anti-TB medications due to fear of sanctions by regulatory bodies. Additionally, this study is cross-sectional, and the actual reasons why the number of apprentices in drug shops is influencing anti-TB stocking and dispensing practices are unknown beyond the associations. Our results, linking the stocking of anti-TB medications to the number of apprentices in a pharmacy, should be interpreted with caution. We did not control for the level of sales in the pharmacies, which may be a more significant factor in determining the stocking of anti-TB medications. Further research is needed to establish a clearer link between the number of apprentices and the stocking of anti-TB, taking into account the potential impact of the level of sales. Therefore, future qualitative studies will need to unravel this. Additionally, future studies need to analyze key issues around supply chain management to ensure a consistent supply of quality-assured products for CPs and PMVs and the impact of stocking non-quality-assured medications on the development of resistance.

## 7. Conclusions

The effectiveness of drug shops depends on the knowledge and experience of their personnel. We propose a CPD-plus approach that requires documented training and on-the-job assessment of apprentices by senior pharmacists and inspection teams. This study, linking the stocking of anti-TB to the number of apprentices, should be viewed with caution due to a lack of control for the level of sales, and future studies should examine the reasons behind the findings and the impact of apprenticeship programs on drug shop operations.

## Figures and Tables

**Table 1:** Sociodemographic characteristics of respondents.

Variables	TOTAL (%)
**Sex**	
Male	243 (60.4%)
Female	159 (39.6%)
**State**	
Lagos	309 (76.3%)
Kebbi	96 (23.7%)
**LGA**	
Agege	37 (9.1%)
Ikeja	5 (1.2%)
Ifelodun Ajeromi	21 (5.2%)
Alimosho	93 (23.0%)
Kosofe	17 (4.2%)
Shomolu	3 (0.7%)
Ojo	64 (15.8%)
Badagry	6 (1.5%)
Apapa	28 (6.9%)
Mushin	9 (2.2%)
Amuwo odofin	11 (2.7%)
Oshodi	5 (1.2%)
Ifako Ijaye	10 (2.5%)
Koko-besse	36 (8.9%)
Argungu	50 (12.3%)
Yauri	10 (2.5%)
**Level of education**	
Primary	7 (1.7%)
Secondary	162 (40.0%)
Tertiary	236 (58.3%)
**Medical education**	
No	105 (25.9%)
Yes	300 (74.1%)
**Specify medical education (N = 201)**	
Patent Med Vendor Tier 1	262 (64.7%)
Tier 2	49 (12.1%)
Tier 3	6 (1.5%)
Comm pharmacist CP	88 (21.7%)
**Years in business**	
0–5 yrs	115 (28.4%)
6 yrs and above	290 (71.6%)
**Trained on TB**	
No	286 (70.6%)
Yes	119 (29.4%)
**Number of apprentices**	
None	130 (32.1%)
1–2	151 (37.3%)
3 and above	124 (30.6%)
**Clients per actual average no. (N=209)**	
1–10	115 (55.0%)
11–20	94 (45.0%)
**Keep any treatment record**	
No	277 (68.4%)
Yes/not seen	72 (17.8%)
Yes/seen	56 (13.8%)
**Referred coughing person in last one month**	
No	209 (51.6%)
Yes	196 (48.4%)
**Kept referral records**	
No	343 (84.7%)
Yes/not seen	39 (9.6%)
Yes/seen	23 (5.7%)
**Willing to be engaged as treatment center for TB**	
No	9 (2.2%)
Yes	396 (97.8%)

**Table 2:** Type of anti-TB medications stocked by CPs and PMVs, n = 68.

Variable	Number/Percentage			
	PPMV	CPs	Total	*p*-value
Rifampicin	33 (53.2%)	29 (46.8%)	62 (100.0%)	0.880
Isoniazid	8 (27.6%)	21 (72.4%)	29 (100.0%)	<0.0001
Pyrazinamide	16 (48.5%)	17 (51.5%)	33 (100.0%)	0.475
Ethambutol	30 (68.2%)	14 (31.8%)	44 (100.0%)	0.001
Streptomycin	28 (58.3%)	20 (41.7%)	48 (100.0%)	0.168

**Table 3:** Bivariate analysis of anti-TB stocking practice and associated factors.

	Does Not Stock	Stock Anti-TB	cOR, 95% CI	*p*-Value	aOR, 95% CI	*p*-Value
**Sex**						
Male	103 (66.5%)	52 (33.5%)	1.32 (0.62–2.81)	0.469		
Female	21 (60%)	14 (40%)				
**State**						
Lagos	59 (60.8%)	38 (39.2%)	0.66 (0.36–1.20)	0.173		
Kebbi	66 (70.2%)	28 (29.8%)				
**Medical education**						
No	45 (71.4%)	18 (28.6%)	1.5 (0.78–2.88)	0.222		
Yes	80 (62.5%)	48 (37.5%)				
**Type of provider**						
PMV	95 (72.5%)	36 (27.5%)	2.64 (1.39–4.98)	0.002	1.03 (0.45–2.33)	0.947
CP	30 (50%)	30 (50%)				
**Years in business**						
0–5 yr	56 (68.3%)	26 (31.7%)	1.25 (0.68–2.29)	0.473		
6 yr and above	69 (63.3%)	40 (36.7%)				
**Prior TB training**						
No	55 (57.9%)	40 (42.1%)	0.32 (0.14–0.73	0.005	0.89 (0.45–1.78)	0.758
Yes	39 (81.2%)	9 (18.8%)				
**Recent ref within 1 month**						
No	61 (67%)	30 (33%)	1.14 (0.63–2.08)	0.66		
Yes	64 (64%)	36 (36%)				
**Willing to be engaged**						
No	1 (100%)	0 (0%)	1.53 (1.38–1.70)	0.466		
Yes	124 (65.3%)	66 (34.7%)				
**Aware of DOTs clinics**						
No	34 (54%)	29 (46%)	0.48 (0.25–0.89)	0.019	1.41 (0.63–3.14)	0.398
Yes	91 (71.1%)	37 (28.9%)				
**Own > 1 store**						
No	114 (69.5%)	50 (30.5%)	3.32 (1.44–7.57)	0.004	0.80 (0.29–2.17)	0.662
Yes	11 (40.7%)	16 (59.3%)				
**Educational qualf**						
Secondary or less	25 (64.1%)	14 (35.9%)	0.93 (0.45–1.94)	0.843		
Tertiary	100 (65.8%)	52 (34.2%)				
**Number of apprentices**						
2 or less	81 (82.7%)	17 (17.3%)	5.31 (2.74–10.29)	<0.001	10.23 (0.10–0.49)	0.001
3 or above	44 (47.3%)	49 (52.7%)				
**Tier class (PMV)**						
Tier 1	61 (68.5%)	28 (31.5%)	0.60 (0.24–1.48)	0.266		
Tier 2 and 3	29 (78.4%)	8 (21.6%)				
**Daily consultation**						
1–20/day	29 (82.9%)	6 (17.1%)	3.02 (1.18–7.71)	0.017	0.45 (0.17–1.24)	0.124
>20/day	96 (61.5%)	60 (38.5%)				
**Kept treatment record**						
No record	113 (65.7%)	59 (34.3%)	1.11 (0.42–2.99)	0.825		
Record seen	12 (63.2%)	7 (36.8%)				
**Kept referral record**						
No record	124 (66.3%)	63 (33.7%)	5.91 (0.60–57.9)	0.086		
Record seen	1 (25%)	3 (75%)				
**Service organization**						
Sell drugs only	0 (0%)	1 (100%)	2.93 (2.40–3.56)	0.168		
Sell drugs and educate clients	125 (65.8%)	65 (34.2%)				

## Data Availability

The data presented in this study are available on request from the corresponding author. The data are not publicly available due to privacy and ethical restrictions.

## Abbreviations

ADDO	Accredited Drug Dispensing Outlets
PMV	Patent Medicine Vendor
CP	Community Pharmacist
DR-TB	Drug-Resistant Tuberculosis
FDC	Fixed-Dose Combination
SPSS	Statistical Package for Social Sciences
DOTS	Directly Observed Treatment Short Course
CI	Confidence Interval
HIV	Human Immunodeficiency Virus
LGA	Local Government Area
CHO	Local Council Development Area
PCN	Pharmacy Council of Nigeria
CHEW	Community Health Extension Workers
NTBLCP	National Tuberculosis and Leprosy Control Program
NTP	National Tuberculosis Program
WHO	World Health Organization

## References

[1] World Health Organization G Global Tuberculosis Report 2021.

[2] Oladimeji O., Othman Y., Oladimeji K.E., Atiba B.P., Adepoju V.A., Odugbemi B.A. (2022). Patterns of Presentation of Drug-Resistant Tuberculosis in Nigeria: A Retrospective File Review. Microbiol. Res..

[3] Oladimeji O., Adeniji-Sofoluwe A.T., Othman Y., Adepoju V.A., Oladimeji K.E., Atiba B.P., Anyiam F.E., Odugbemi B.A., Afolaranmi T., Zoakah A.I. (2022). Chest X-ray Features in Drug-Resistant Tuberculosis Patients in Nigeria; a Retrospective Record Review. Medicines.

[4] World Health Organization (2018). Global tuberculosis Report. https://apps.who.int/iris/handle/10665/274453.

[5] Ukwaja K.N., Alobu I., Nweke C.O., Onyenwe E.C. (2013). Healthcare-seeking behavior, treatment delays and its determinants among pulmonary tuberculosis patients in rural Nigeria: A cross-sectional study. BMC Health Serv. Res..

[6] Aniebue P., Onoka C. (2009). Delay in healthcare seeking for treatment amongst patients with pulmonary tuberculosis in Enugu, South-East Nigeria. Int. J. Med. Health Dev..

[7] Bames J., Chandani T., Feeley R. (2008). Nigeria Private Sector Health Assessment-Private Sector Partnership One Project.

[8] Pharmacists Council of Nigeria. https://www.pcn.gov.ng/pharmacy-practice/.

[9] De La Cruz A., Liu J., Schatzkin E., Modrek S., Oladepo O., Dominic M. The Influence of policy, hierarcy, and competition on malaria case management among retail providers in Nigeria. Proceedings of the American Society for Tropical Medicine and Hygiene 61st Annual Meeting.

[10] Lambert M.L., Delgado R., Michaux G., Volz A., Van der Stuyft P. (2004). Tuberculosis control and the private health sector in Bolivia: A survey of pharmacies. Int. J. Tuberc. Lung Dis..

[11] Newell J., Pande S., Baral S., Bam D., Malla P. (2004). Control of tuberculosis in an urban setting in Nepal: Public-private partnership. Bull. World Health Organ..

[12] Kobaidze K., Salakaia A., Blumberg H. (2009). Over the Counter Availability of Antituberculosis Drugs in Tbilisi, Georgia in the Setting of a High Prevalence of MDR-TB. Interdiscip. Perspect. Infect. Dis..

[13] Satellite View and Map of the city of Lagos. https://www.nationsonline.org/oneworld/map/google_map_Lagos.html.

[14] Monsudi K.F. (2011). Impact of Cataract Surgery on Visual Function and Quality of Life in Birnin Kebbi, Nigeria Part II.

[15] Federal Republic of Nigeria: 2006 Population Census. http://www.nigerianstat.gov.ng/Connections/Pop2006.pdf.

[16] OlaOlorun F.M., Jain A., Olalere E., Daniel-Ebune E., Afolabi K., Okafor E., Dwyer S.C., Ubuane O., Akomolafe T.O., Baruwa S. (2022). Nigerian stakeholders’ perceptions of a pilot tier accreditation system for Patent and Proprietary Medicine Vendors to expand access to family planning services. BMC Health Serv. Res..

[17] Obi I.E., Nwagbo D., Onwasigwe C.N. (2010). Tuberculosis knowledge, perception and practice among patent medicine vendors in south-east, Nigeria. J. Coll. Med..

[18] Onwuka E. How to Start/Open Chemist (Patent Medicine) Drug Store in Nigeria. https://www.naijaonlinebiz.com/how-to-start-open-new-chemist-drug-store-in-nigeria/comment-page-1/.

[19] Oyeyemi A.S., Ogunnowo B.E., Odukoya O.O. (2014). Patent medicine vendors in rural areas of Lagos Nigeria: Compliance with regulatory guidelines and implications for malaria control. Trop. J. Pharm. Res..

[20] Akuse R.M., Eseigbe E.E., Ahmed A., Brieger W.R. (2010). Patent Medicine Sellers: How Can They Help Control Childhood Malaria?. Malar. Res. Treat..

[21] Adibe M.O., Ayogu E.E., Igboeli N.U. (2016). Assessment of knowledge and roles of patent medicine vendors in the implementation of national malaria treatment policy in Nigeria. World J. Pharm. Sci..

[22] Pacqué M., Gilroy K. Findings from the Enhancing Quality iCCM through Proprietary and Patent Medical Vendors (PPMV) and Partnerships (EQuiPP) Approach. https://www.childhealthtaskforce.org/sites/default/files/2019-08/MCSP%20EQuiPP%20%28PSE%20Subgroup%20Meeting%29_07.30.2019.pdf.

[23] Baruwa S., Tobey E., Okafor E., Afolabi K., Akomolafe T.O., Ubuane I., Anyanti J., Jain A. (2022). The role of job aids in supporting task sharing family planning services to community pharmacists and patent proprietary medicine vendors in Kaduna and Lagos, Nigeria. BMC Health Serv. Res..

[24] Agyepong I.A., Ansah E., Gyapong M., Adjei S.K., Barnish G., Evans D.B. (2002). Strategies to improve adherence to recommended chloroquine treatment regimens: A quasi-experiment in the context of integrated primary health care delivery in Ghana. Soc. Sci. Med..

[25] Bradley F., Schafheutle E.I., Ma S.C.W., Noyce P.R. (2013). Changes to supervision in community pharmacy: Pharmacist and pharmacy support staff views. Health Soc. Care Community.

[26] Gernant S.A., Nguyen M.-O., Siddiqui S., Schneller M. (2018). Use of pharmacy technicians in elements of medication therapy management delivery: A systematic review. Res. Soc. Adm. Pharm..

[27] Muirhead V., D’Antoni D., Auyeung V. (2020). Community pharmacy staff oral health training, training needs and professional self-efficacy related to managing children’s dental problems. Int. J. Pharm. Pr..

[28] Thomas C.E.L., Phipps D.L., Ashcroft D.M. (2016). When procedures meet practice in community pharmacies: Qualitative insights from pharmacists and pharmacy support staff. BMJ Open.

[29] Donovan G., Paudyal V. (2015). England’s Healthy Living Pharmacy (HLP) initiative: Facilitating the engagement of pharmacy support staff in public health. Res. Soc. Adm. Pharm..

[30] Improving Access to Essential Medicine in Tanzania. https://www.pc.go.tz/files/The%20ADDO%20Program%20%20Story-%20Feb%202015.pdf.

